# Chronic Traumatic Encephalopathy and Suicide: A Systematic Review

**DOI:** 10.1155/2013/424280

**Published:** 2013-11-17

**Authors:** Hal S. Wortzel, Robert D. Shura, Lisa A. Brenner

**Affiliations:** ^1^Departments of Psychiatry, Neurology, and Physical Medicine and Rehabilitation, Veterans Integrated Service Network (VISN) 19 Mental Illness, Research, Education and Clinical Center, Denver Veterans Affairs Medical Center, Denver, CO 80220, USA; ^2^Departments of Psychiatry and Neurology, University of Colorado School of Medicine, Denver, CO 80045, USA; ^3^Department of Psychology, Marshall University, Huntington, WV 25755, USA

## Abstract

Traumatic brain injury (TBI) is a global health concern, and the recent literature reports that a single mild TBI can result in chronic traumatic encephalopathy (CTE). It has been suggested that CTE may lead to death by suicide, raising important prevention, treatment, and policy implications. Thus, we conducted a systematic review of the medical literature to answer the key question: *What is the existing evidence in support of a relationship between CTE and suicide?* Systematic searches of CTE and suicide yielded 85 unique abstracts. Seven articles were identified for full text review. Only two case series met inclusion criteria and included autopsies from 17 unique cases, 5 of whom died by suicide. Neither studies used blinding, control cases, or systematic data collection regarding TBI exposure and/or medical/neuropsychiatric history. The identified CTE literature revealed divergent opinions regarding neuropathological elements of CTE and heterogeneity regarding clinical manifestations. Overall quality of evidence regarding a relationship between CTE and suicide was rated as very low using Grading of Recommendations Assessment, Development and Evaluation Working Group (GRADE) criteria. Further studies of higher quality and methodological rigor are needed to determine the existence and nature of any relationship between CTE and suicide.

## 1. Introduction

The potential consequences of repetitive brain trauma have long been recognized, dating back to the initial description of “punch drunk” boxers by Martland in 1928 [1], subsequently referred to as dementia pugilistica in the medical literature. More recently, the term chronic traumatic encephalopathy (CTE) has been introduced to describe progressive neuropathological and clinical manifestations associated with traumatic brain injury (TBI). However, unlike the initial descriptions of this neuropsychiatric phenomenon, involving chronic traumatic brain injury sustained over boxing careers, the more recent literature describing CTE suggests that even a single (mild) TBI may yield a progressive and devastating neurodegenerative illness [2, 3], thereby expanding the at-risk population to many millions. Adding to consternation, and complicating the identification of causal relationships, are reports suggesting that the clinical symptoms of CTE may be delayed in onset, emerging as far out as 8–10 years following TBI exposure [4, 5]. The recent CTE literature also expands the clinical phenomenology of this proposed neuropsychiatric condition, including explicit associations with suicide. McKee et al. recently described the “spectrum of disease” in CTE: “CTE is clinically associated with symptoms of irritability, impulsivity, aggression, depression, short-term memory loss and heightened suicidality” [5].

A number of case reports and/or case series describe suicide deaths and/or behaviors that have followed exposure to TBI and are correlated with histopathology that is reportedly consistent with CTE [6–11]. Such reports, involving both professional athletes and United States Veterans, have generated considerable media attention and widespread public concern regarding a possible connection between CTE and suicide [12, 13]. A number of publications from the popular press feature statements suggesting rather strong relationships between CTE and suicide deaths. A New York Times article following the suicide death of NFL player Dave Duerson reports “indisputable evidence of CTE” and “no evidence of any other disorder” [14]. A more recent article, also featured in The New York Times, reports upon recent findings of CTE in United States Veterans of the wars in Iraq and Afghanistan, posing the question “Could blast from bombs or grenades have a catastrophic impact similar to those of repeated concussions in sports, and could the rash of suicides among young veterans be a result?” Reportedly, at least one CTE expert's hypothesis is that CTE accounts for a share—he has no idea how large—of veteran suicides [15].

The supposition of a relationship between CTE and death by suicide has significant implications pertaining to clinical practice, public safety and health, and policy making for the Departments of Defense and Veterans Affairs and professional sports organizations, as well as the potential for medicolegal repercussions. In light of the gravity of such implications, and the millions of individuals potentially at risk, a critical review regarding the state of the science linking CTE and suicide is necessary. Hence, we have conducted a systematic review of the existing medical literature describing CTE and suicide with the following key question in mind: *What is the existing evidence in support of a relationship between CTE and suicide?*

## 2. Methods

### 2.1. Search Strategy

PubMed was searched on January 3, 2013 for peer-reviewed articles published in English, without restriction to date/year of publication, and involving human subjects to identify articles germane to the key research question listed above. The primary search strategy included a systematic and comprehensive search for suicide and chronic traumatic encephalopathy terms (see Table 1). Similar searches were performed in PsycINFO and EMBASE databases, with analogous strategies employed. Additional searches were conducted in Google Scholar, Google, and EBSCOhost (Medline, Academic Search Premier, and PsycARTICLES) using the terms “chronic traumatic encephalopathy and suicide.” References listed in identified publications were reviewed for additional possible articles for inclusion. Identified subject-matter experts in TBI, CTE, and suicide were contacted via e-mail, provided with the list of identified publications for inclusion, and asked to submit suggestions for additional articles for consideration; no recommendations for additional publications were received.

### 2.2. Study Selection

All identified abstracts were independently reviewed by two of the authors. Abstracts (*n* = 85) were randomly assigned. Reviewers were provided with Microsoft Access 2007 databases preloaded with assigned abstracts, which allowed for reviewers to remain blind to each other's reviews. Criteria for inclusion for full review included: (1) full-text article in English; (2) adult or mixed adult/child population; (3) met the case definition for CTE (“chronic cognitive and neuropsychiatric symptoms of chronic neurodegeneration following a single episode of severe traumatic brain injury or repeated episodes of mild traumatic brain injury; CTE can only be definitively diagnosed by direct tissue examination” [7]); (4) met the case definition for suicide as an outcome (“death caused by self-inflicted injurious behavior with any intent to die as a result of the behavior” [16]); (5) was a peer-reviewed journal article which contained original research. In cases of discrepancies between reviewers, the third reviewer made the tie-breaking decision for inclusion/exclusion. 

**Figure 1:**
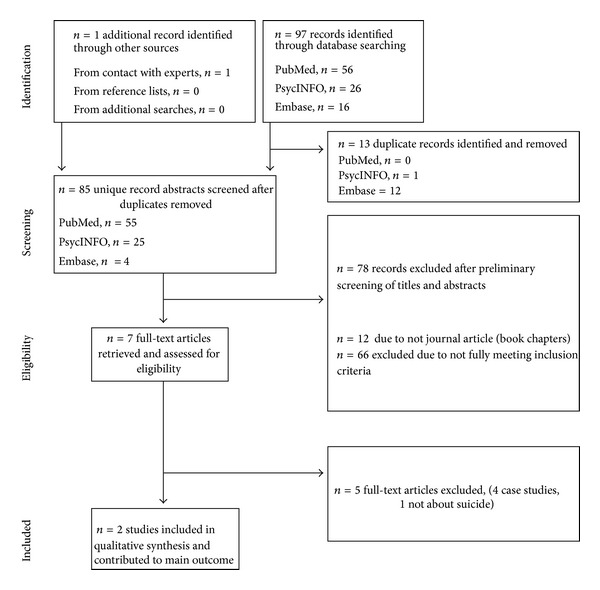
PRIMSA flow diagram.

For the full article review phase (*n* = 7), Access databases were once again created for each reviewer. The inclusion criteria described above were reapplied. All articles were reviewed by all three reviewers and coded independently. In cases in which determination to exclude was not unanimous, consensus was arrived at by discussion between all three reviewers. Of note, original exclusion criteria (single case study; nonconsecutive or nonrandom case series; selected samples or studies not reporting a random or consecutive sample; study sample size of greater than 30) resulted in elimination of all identified publications. Consequently, the decision was made to extend review to nonconsecutive case series, thereby identifying two studies for inclusion. The literature flow is illustrated in the Preferred Reporting Items for Systematic Reviews and Meta-Analyses (PRISMA) Flow Diagram (Figure 1). In the end, two articles met the more liberal inclusion criteria. 

### 2.3. Quality of Evidence

Given the nature of the identified literature, review of individual articles was conducted using the New Castle-Ottawa Quality Assessment Scale for Observational Studies [17]. Risk of bias was assessed per Agency for Healthcare Research and Quality (AHRG) [18] recommendations. Studies meeting inclusion criteria following full-text review were evaluated with respect to the overall quality of the evidence, which was evaluated using the criteria recommended by the Grading of Recommendations Assessment, Development and Evaluation Working Group (GRADE) [19]. 

## 3. Results and Discussion

As illustrated in the PRIMSA flow diagram (Figure 1), of the 85 unique records identified, only seven publications met criteria for full-text review (Table 2). Of these, four were excluded as single case studies, and an additional publication that was excluded as suicide was not a specific outcome. This left two case series. The first of these [7] presented five cases involving professional American athletes of contact sports that engaged in “parasuicide and suicides.” Although all of the brains examined appeared grossly normal on examination, they all reportedly featured histopathological evidence of CTE. The second series [3] presented cases involving fourteen profession athletes and three high school football players. The authors reported an extraordinarily broad (and nonspecific) “emerging syndromic profile” for CTE including the following features: premorbid history of amateur or professional participation in contact sports; long latent period between initial play and symptom manifestation; progressive deterioration in social and cognitive functioning (manifesting as loss of memory, loss of language, loss of executive function as evidenced by dismal business/investment performance, dismal money management, deterioration in socioeconomic status, and/or bankruptcy); mood and behavioral disorders, including paranoid ideation, social phobia, exaggerated response to life stressors, bouts of anger and/or agitation, rampant fluctuations in mood, major depression, suicidal ideation, suicidal behavior, insomnia, and/or hyperactivity; progressive deterioration in interpersonal or familial relationships; criminal and/or violent tendencies and behaviors; substance abuse, including alcohol, prescription drugs, and illicit drugs; increasing religiosity; headaches, generalized body aches, and pain. Diffuseness of the reported findings extended to histopathology; the authors reported four emerging histopathological variants of CTE despite the modest number of total cases. Five of the 17 reported cases involved death by suicide.

In reviewing this literature, it is notable that some individual CTE cases appear to be presented repeatedly in both single case report form and again in subsequent case series; in some instances individual cases were represented twice. For example, a CTE case involving the suicide death of a professional wrestler was first presented in a case report [10] and then appears to have been subsequently included in 2 case series [3, 7]; explicit indication of the representation of individual CTE cases was not always apparent in these publications; thereby necessitating careful review across the publications to determine the actual number of individual CTE cases and suicide deaths was presented. Such a review across the publications listed in Table 1 revealed that a total of only 21 unique CTE cases were presented, only five of which involved deaths by suicide (four athletes and one Veteran). Among those five individual cases involving death by suicide, review across presentation in both case reports and case series revealed a number of suicide risk factors (e.g., substance use/abuse, depression, chronic pain, male gender, psychosocial stressors); analysis and/or commentary regarding the relative weight/contribution of various risk factors to suicide death was lacking. Additionally, it is not apparent that any systemic or uniform procedure for identifying TBI exposure history and/or all pertinent suicide risk factors was employed. 

The two articles were reviewed with the New Castle-Ottawa Quality Assessment Scale for Observational Studies [17]. Regarding case selection and representativeness of the exposed cohort, the nonconsecutive cases selected for inclusion involve professional athletes and feature a multitude of neuropsychiatric comorbidities (and suicide risk factors), such that these cases are not representative of the vast majority of individuals in the general community with history of TBI and/or theoretically at risk for CTE. It is not apparent if independent validation or record linkage was employed to confirm cases of suicide. For example, case no. 4 in [7] involved death from a motor vehicle accident during a high-speed police chase that was categorized as a suicide death in a subsequent case series [3], although the method for establishing death by suicide is not explicated. Regarding comparability, no study controls were employed. Regarding outcome, no independent blind assessment for CTE findings was employed. Follow-up considerations are not applicable in light of the nature of the cohorts (all deceased).

A separate review to assess for the risk of bias across these studies, anchored to criteria/definitions endorsed by the AHRQ [18], suggested a high risk of bias for both publications. In the first of the above described case series, all individual cases were selected specifically because they featured self-directed violent behavior (almost all resulting in death by suicide), suggesting prominent neuropsychiatric comorbidity, and creating obvious risk in terms of selection bias. Given the nonrandomized and nonconsecutive nature of case selection across these two case series, the lack of blinding surrounding histopathological analysis and/or exposure history, and lack of systematic and/or operationalized assessment for neuropsychiatric comorbidity, performance bias, detection bias, and/or potential confounding factors all threaten the validity of the findings reported, in terms of purported relationships between TBI exposure history and histopathological findings, and between histopathological features and instances of self-directed violence. 

GRADE criteria [19] were applied to the body of evidence with suicide being the outcome of interest. The two identified case series resulted in an initial quality of evidence rating of low based simply upon the observational nature of the investigations to date. However, additional considerations surrounding the methodologies employed, potential for bias, and confounding factors mandated downgrading of the quality of evidence to very low. 

## 4. Conclusions

Returning to our key question, *what is the existing evidence in support of a relationship between CTE and suicide*, systematic review of the applicable literature and application of established methods for rating quality of evidence indicates scant evidence in support of such. This process currently yields only 2 case series, which collectively offer a very low quality of evidence and high risk of bias. Although absence of proof is admittedly not the same as proof of absence, the current state of the science indicates that suppositions invoking a relationship between CTE and suicide must be viewed as speculative at this point in time. 

Although not a study on CTE *per se*, a recent publication by Baron et al. [20] is notable for reporting overall reduced mortality among retired NFL players relative to the general population, and reduced mortality from suicide in particular. In that publication, the calculated expected number of deaths from suicide was 21.8, the actual observed number of suicide deaths was 9, and the standardized mortality ratio for suicide was 0.41 (95% confidence interval of 0.19–0.78). Such findings strongly run counter to theories positing that NFL players are at high risk from suicide secondary to high rates of CTE resulting from high rates of multiple TBIs. If the latter portion of that theory is correct (exposure to multiple TBIs yields high rates of CTE), then the relatively low rates of suicide observed among retired NFL players would suggest relatively weak associations between CTE and deaths by suicide. 

Granier et al. [21] caution against “leaping too far too soon” regarding our present understanding of CTE, posing the question, “given the state of the literature, why have the prevailing winds of popular culture shifted toward acceptance of a causal linked between concussion and CTE without valid scientific evidence of such a link?” In answering their own question, those authors observe media sensationalism and financial pressures potentially influencing public opinion on such matters. In light of the results from the current systematic review, a similar question regarding the widespread acceptance of a causal link between CTE and suicide begs consideration.

The current state of science surrounding CTE generally warrants considerable modesty regarding our current understanding of any clinical phenomenology associated with the histopathological entity termed CTE, and clearly precludes any claims suggesting evidence-based relationships between CTE and suicide. Hence, individual cases of suicide continue to mandate case-by-case analysis regarding the relative contributions of various surrounding neuropsychiatric and psychosocial factors. Histopathological findings of CTE at this point in time are likely to provide little incremental value to such determinations, such that formulations regarding the relative contribution of neurotrauma to a given case of suicide are probably better informed by a far more developed literature describing the association between TBI and suicide more generally [22–24]. Well-designed, prospective, longitudinal, large-population studies are required to determine the existence and nature of any possible (and sure to be complex) relationships between concussions, CTE, and death by suicide.

## Figures and Tables

**Table 1:** PubMed search strategy.

(1) Suicide[MeSH]—44,511 results	
(2) Suicide, attempted[MeSH]—13,881 results	
(3) Attempted suicide OR suicide prevention OR suicide risk OR suicide self-regulation OR suicidal ideation OR suicid* behaviour OR suicid* behavior OR suicidality OR suicid* OR self injur* OR self-injur* OR self harm OR self-harm OR parasuicid* OR deliberate self injur* OR deliberate self-injur* OR deliberate self harm OR deliberate self-harm OR self directed violen* OR self-directed violen*—70,747 results	
(4) (#1) OR (#2) OR (#3)—70,747 results	
(5) Chronic traumatic encephalopathy OR dementia pugilistica—6,407 results	
(6) (#4) AND (#5)—62 results	
(7) Limit (#6) to English—56 results	

**Table 2:** Full articles reviewed.

Authors	Year	Journal	Population	Age	*n *	Profession	Type	Design	Death
Omalu et al. [6]	2011	Neurosurg Focus	Adult	27	1	Military	OBS	CSt	S: hanging
Omalu et al. [3]	2011	Neurosurgery	Mixed	16–52	17	Mixed athletes	OBS	CSe	S (×5): mixed
Omalu et al. [7]	2010	Am J Forensic Med Pathol	Adult	36–50	5	NFL ×4, wrestler ×1	OBS	Cse	S (×5): mixed
Omalu et al. [10]	2010	Journal of Forensic Nursing	Adult	40	1	Wrestler	OBS	CSt	S: hanging
Omalu et al. [10]	2010	Journal of Forensic Nursing	Adult	44	1	NFL	OBS	CSt	S: GSW
McKee et al. [4]	2009	Neuropathol Exp Neurol	Adult	23–91	3	Mixed	OBS	CSe	Unknown
Omalu et al. [8]	2006	Neurosurgery	Adult	45	1	NFL	OBS	CSt	S: poisoning

Note. OBS: observational study; CSt: single case study; Cse: case series; S: death by suicide; GSW: gun shot wound.
